# Procedural outcomes and learning curve of cardiac arrhythmias catheter ablation using remote magnetic navigation: Experience from a large‐scale single‐center study

**DOI:** 10.1002/clc.23391

**Published:** 2020-05-26

**Authors:** Xiang Li, Qi Jin, Ning Zhang, Tianyou Ling, Changjian Lin, Kangni Jia, Yangyang Bao, Yun Xie, Yue Wei, Kang Chen, Wenqi Pan, Yucai Xie, Liqun Wu

**Affiliations:** ^1^ Department of Cardiology, Shanghai Ruijin Hospital Shanghai Jiao Tong University School of Medicine Shanghai China

**Keywords:** arrhythmia, catheter ablation, learning curve, remote magnetic navigation

## Abstract

**Background:**

Remote magnetic navigation (RMN)‐guided ablation has become an inspiring method of catheter ablation for tachyarrhythmias.

**Hypothesis:**

Data from a large‐scale single center may provide further insight into the safety of and the learning curve for RMN‐guided ablation.

**Methods:**

A total of 1003 catheter ablation procedures using RMN for conditions including supraventricular ventricular tachycardia, atrial tachyarrhythmias, and premature ventricular contraction/ventricular tachycardia (PVC/VT) were retrospectively analyzed from an ablation registry. Procedural outcomes, including procedure time, mapping time, X‐ray time, and RF time, were assessed. The complications were classified into two categories: major and minor. A subanalysis was used to illustrate the learning curve of RMN‐guided ablation by assessing procedure time and total X‐ray time of 502 atrial fibrillation (AF) ablation procedures.

**Results:**

Among these procedures, 556 (55.4%) were AF and 290 (28.9%) were PVC/VT. Electrical pulmonary vein isolation was achieved in 99.0% of AF procedures, and acute success reached 90.3% in PVC/VT procedures. The overall complication rate was 0.5%. In the subanalysis of AF procedures, the overall procedure time and X‐ray time of procedures were short (125.9 ± 54.6 and 5.3 ± 3.9 minutes, respectively) and proceeded to decrease from the initial 30 procedures to about 300 procedures, where the learning curve reached plateau, demonstrating maximum procedure efficiency.

**Conclusions:**

RMN‐guided ablation is safe, as verified by very low overall complication rate and reduced X‐ray time. In our study, even the first AF procedures had a relatively low procedure time and total X‐ray time, and procedure efficiency improved during the learning curve.

## 1 INTRODUCTION

Catheter ablation has been well established in the treatment of a variety of tachyarrhythmias.^1^ Such an anatomy‐based therapy has placed high requirements on the precision of localization, stability of catheter contact, and catheter flexibility. Even experienced cardiac electrophysiologists performing certain ablation procedures may encounter difficulties in reaching complex anatomical regions and maintaining the stability of the catheter, leading to impaired safety and success rate.^2^, ^3^, ^4^ The remote magnetic navigation (RMN) system for catheter ablation offers the advantages of precise and flexible catheter navigation,^5^, ^6^ reduction in peri‐procedure complications,^7^ and X‐ray exposure.^8^ However, previous studies regarding RMN‐guided ablation have been limited by small sample size and few arrhythmia types. In addition, no large sample data targeted the analysis of a learning curve for procedure efficiency. Here, we have studied over 1000 RMN‐guided ablation cases in a single center, with the objectives of evaluating its safety and the procedural outcomes among patients with different types of arrhythmias, and determining the learning curve for RMN‐guided ablation.

## 2 METHODS

### 2.1 Patient population

Between May 2010 and October 2019, 1003 consecutive cases, ranging from supraventricular tachycardia (SVT) to atrial tachycardia (AT), atrial flutter (AFL), atrial fibrillation (AF), premature ventricular contraction (PVC), and ventricular tachycardia (VT), targeted for ablation were included in a registry at Ruijin Hospital, Shanghai Jiao Tong University School of Medicine. The diagnoses of patients included in the registry were all established according to the current guidelines.^9^, ^10^, ^11^ All the patients signed informed consent prior to the RMN‐guided ablation procedure. Any antiarrhythmic drug was discontinued before the ablation procedure for at least 5 half‐lives except the beta‐blockers for the control rate for persistent AF and VT storm.

### 2.2 Preparation for procedure

For patients with SVT, internal jugular and left femoral veins were selected for puncture. A decapolar catheter, a bipolar catheter, and a quadripolar catheter (St. Jude Medical, Inc., St. Paul, Minnesota) were placed within the coronary sinus, at the right ventricle apex, and at the His bundle, respectively.

For patients with AF, standard anticoagulation therapy was given prior to the procedure. All patients underwent effective anticoagulation therapy for at least 1 month prior to the procedure, either with an uninterrupted vitamin‐K antagonist (warfarin) or with non‐vitamin‐K antagonist oral anticoagulants, dabigatran, or rivaroxaban. INR was not allowed to exceed 3 prior to ablation for the patients administered with warfarin. Preprocedural transesophageal echocardiography was used to exclude atrial thrombosis. A decapolar catheter and a bipolar catheter (St. Jude Medical, Inc., St. Paul, Minnesota) were placed within the coronary sinus and at the right ventricle apex, respectively. Conscious sedation was achieved by intravenous injection of midazolam and fentanyl.

For patients with ventricular arrhythmias, a decapolar catheter and a bipolar catheter (St Jude Medical, Inc., St. Paul, Minnesota) were placed, if necessary, within the coronary sinus and at the right ventricle apex, respectively.

### 2.3 Mapping and ablation with RMN

**FIGURE 1:**
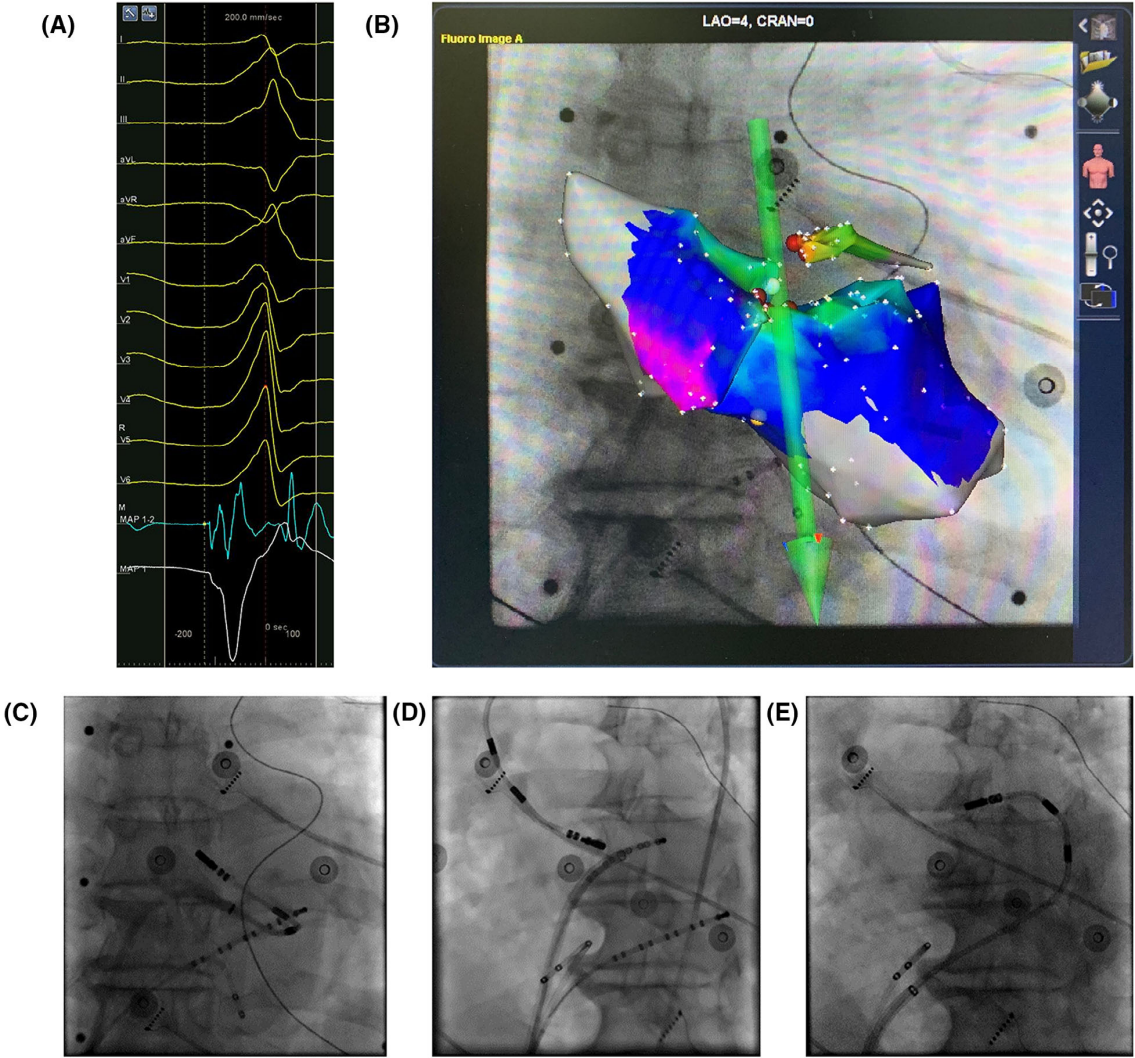
An example of mapping and ablation for premature ventricular contraction (PVC) using remote magnetic navigation (RMN). A, Surface ECG of PVC and intracardiac recordings of the earliest activation site in coronary sinus (great cardiac vein). B, The combination of activation CARTO map of PVC and X‐ray image in “Navigant” system. C, Fluoroscopic view in the posterior‐anterior oblique view. The ablation catheter is pointing toward LV summit after reaching LV cavity through a transseptal approach, this forming a “reverse S‐curve.” D, Fluoroscopic view in the left anterior oblique view. The ablation catheter is pointing toward left coronary cusp through a retrograde transaortic approach. E, The ablation catheter was manipulated with RMN in the great cardiac vein. The catheter tip is equipped with three small magnets. By changing the relative orientation of the external magnets, effective control of the catheter deflection can be achieved

The RMN Niobe ES (Stereotaxis Inc., St. Louis, Missouri), the CARTO 3D mapping system (Biosense Webster Inc., Carlsbad, California), and an open irrigated magnetic ablation catheter (NaviStar RMT ThermoCool; Biosense Webster Inc.) mainly constitute the magnetic navigation system for 3D electroanatomic mapping and remote ablation (Figure 1). Briefly, two permanent magnets are located on two sides of the radiological examination table. When the magnets are in the navigation position, a uniform magnetic field of 0.08 T can be created within the patient's chest. By changing the relative orientation of the magnets, effective control of the catheter deflection can be achieved.

### 2.4 Definitions of procedural outcomes

Procedure time was defined as the total time from the Navigant “open procedure” to the Navigant “close procedure” (in minutes). Clinical start time was the time at which the catheter registered in the CARTO system or the time of first applied magnetic field, whichever time was earlier. Clinical time was calculated as the time difference between clinical start time and the time of last applied field or last RF ablation going off, whichever time is later. Mapping time was the time interval from clinical start time to first burn. Total X‐ray time and the control room's X‐ray time were defined as the total sum of the number of minutes the fluoroscopy beam was activated and when the fluoroscopy beam was activated while magnets were in a navigate position, respectively. Doctor's X‐ray time was calculated as the time difference between the total X‐ray time and the control room's X‐ray time. RF applications and RF time reflected the total sum of the number and minutes of ablation burns in the procedure, respectively.

Acute procedural success was the endpoint of the study. Generally, for SVT and AT, at the end of the procedure, noninducibility of the tachycardias before and after giving isoproterenol was defined as acute success. For typical AFL, acute success was defined as noninducibility of the tachycardias and bidirectional block. For AF patients, acute success was defined as complete electrical pulmonary vein isolation (PVI). For PVC/VT patients, acute ablation success was defined as the elimination and noninducibility of clinical PVC/VT with isoproterenol infusion after at least a 30‐minutes waiting period.

### 2.5 Learning curve

We divided consecutive AF procedures performed by one operator into three phases for analyzing the learning curve with two parameters of procedural outcomes: procedure time and X‐ray time. The cutoff points between the first phase (P1) and the second phase (P2) depended on where the learning curve started to drop significantly. The point where the curve flattened divided subsequent procedures into the P2 and the third phase (P3).

### 2.6 Complications

Adverse events or peri‐procedural complications in this study were divided into two categories: major and minor. Major complications consisted of cardiac tamponade, severe pulmonary vein stenosis, atrial‐esophageal fistula, permanent phrenic nerve palsy, stroke, acute myocardial infarction, permanent heart block, major bleeding, and peri‐procedural death. Minor complications contained vascular complications, transient ischemic attack, temporary heart block, and minor bleeding.

### 2.7 Statistical analysis

Statistical analysis was performed using the Statistical Analysis System 9.3 (SAS, SAS Institute Inc.). Measurement data were expressed as mean ± SD and enumeration data as absolute number and percentages. For intergroup comparison, Welch's *t* test or Mann‐Whitney test was used. Categorical variables were assessed by Pearson's chi‐square test or the Fisher exact test where appropriate. Kruskal‐Wallis test was used for comparison of nonnormally distributed data of several groups. The least absolute residuals method was used for spline fit. The duration of learning curve was determined by linear regression. The first cutoff point is identified by the point where the slope begins to deviate significantly from zero while performing linear regression on data prior to the point. The second cutoff point is identified by the point where the slope begins to be zero while performing linear regression on data after the point. A *P*‐value <.05 was taken as statistical significance.

## 3 RESULTS

### 3.1 Distribution of the procedures

**FIGURE 2:**
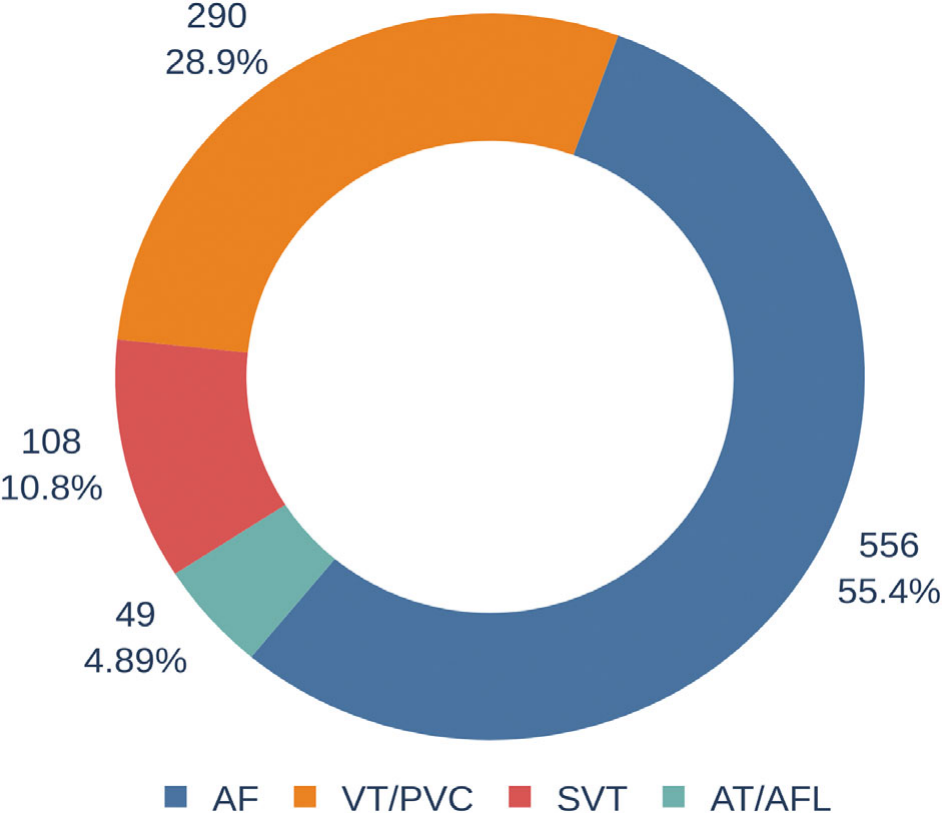
Types of cardiac arrhythmias distribution of 1003 procedures: 108 (10.8%) were supraventricular tachycardia (SVT), 49 (4.9%) were atrial tachycardia (AT) or atrial flutter (AFL), 556 (55.4%) were atrial fibrillation (AF), and 290 (28.9%) were premature ventricular contraction (PVC) or ventricular tachycardia (VT)

Overall, 1003 procedures were enrolled in the study. Among these procedures, 108 (10.8%) were SVT, 49 (4.9%) were AT/AFL, 556 (55.4%) were AF, and 290 (28.9%) were PVC/VT (shown in Figure 2).

### 3.2 Complications

A detailed description of the complications is given in Table 1. One major complication occurred, which was a cardiac tamponade observed in an AF patient. We performed pericardiocentesis and drainage, and after echocardiography demonstrated no intrapericardial bleeding, the drainage tube was removed. The cardiac tamponade was detected after the ablation procedure and might be attributable to a perforation by the coronary sinus catheter. Vascular complications occurred in four cases and were considered minor complications. No procedure‐related death occurred. The overall complication rate was 0.5%, with 0.1% major and 0.4% minor complication rates. There was no significant peri‐procedural complications difference among ablations for supraventricular, atrial, and ventricular arrhythmias (*P* = .62).

**TABLE 1:** Complications: ablation for cardiac arrhythmias using RMN

	Total	SVT	AT/AFL/AF	PVC/VT
	n = 1003	n = 108	n = 605	n = 290
Major total	1 (0.1%)	0	1 (0.2%)	0
Cardiac tamponade	1	0	1	0
Severe PV stenosis	0	—	0	—
AE fistula	0	—	0	—
Permanent PNP	0	0	0	—
Stroke	0	0	0	0
AMI	0	0	0	0
Permanent heart block	0	0	0	0
Major bleeding	0	0	0	0
Death	0	0	0	0
Minor total	4 (0.4%)	1 (0.9%)	2 (0.3%)	1 (0.3%)
Vascular complications	4	1	2	1
TIA	0	0	0	0
Temporary heart block	0	0	0	0
Minor bleeding	0	0	0	0
Total*	5 (0.5%)	1 (0.9%)	3 (0.5%)	1 (0.3%)

Abbreviations: AE, atrial‐esophageal; AF, atrial fibrillation; AFL, atrial flutter; AMI, acute myocardial infarction; AT, atrial tachycardia; PNP, phrenic nerve palsy; PVC, premature ventricular contraction; RMN, remote magnetic navigation; SVT, supraventricular tachycardia; TIA, transient ischemic attack; VT, ventricular tachycardia.

*P = .62.

### 3.3 Procedural outcomes

**FIGURE 3:**
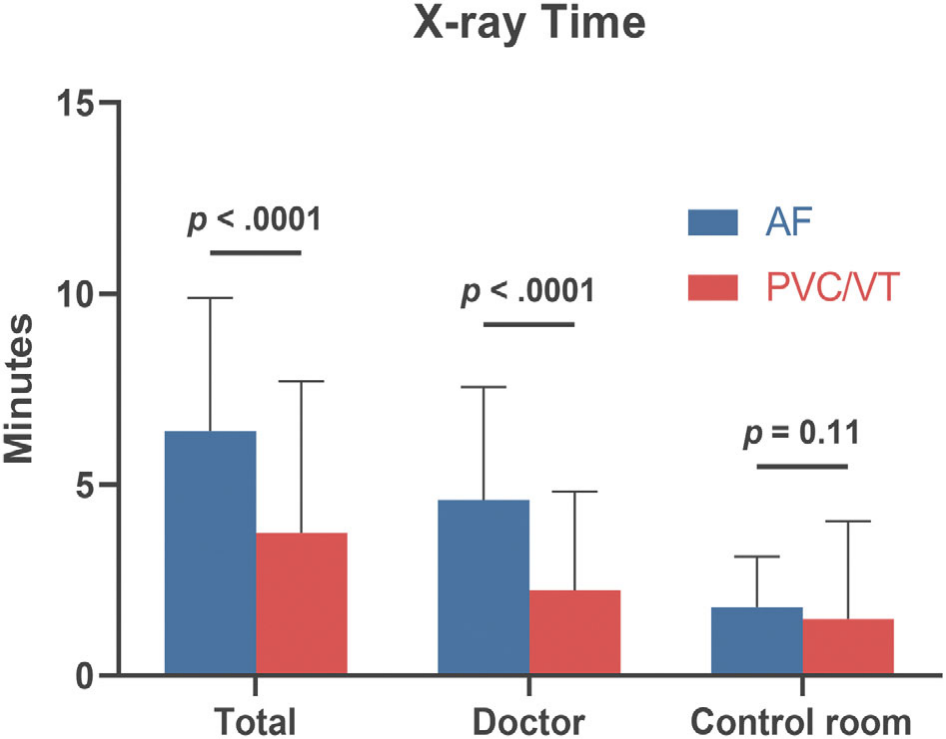
X‐ray time comparisons between atrial fibrillation (AF) and premature ventricular contraction/ventricular tachycardia (PVC/VT) group. Total: total X‐ray time; doctor: doctor's X‐ray time; control room: control room's X‐ray time

Procedural outcomes data included 821 complete record sets that were automatically derived from the RMN system (Table 2) from May 2015. Procedural outcomes data sets prior to 2015 were incomplete and were not included in this analysis. The overall procedural, clinical, and mapping time were 125.9 ± 54.6, 88.9 ± 42.6, and 17.7 ± 11.1 minutes, respectively. Total X‐ray and doctor's X‐ray times were 5.3 ± 3.9 and 3.7 ± 3.1 minutes, respectively. RF applications and RF time were 51 ± 37 and 29.3 ± 19.2 minutes, respectively. We observed significant differences among these groups (each *P* < .0001). Total X‐ray time and doctor's X‐ray time of AF and PVC/VT groups were significantly different (6.4 ± 3.5 vs 3.7 ± 4.0 minutes, *P* < .001; 4.6 ± 3.0 vs 2.2 ± 2.6 minutes, *P* < .001), while no significant difference was observed in control's room X‐ray time (1.8 ± 1.3 vs 1.5 ± 2.6 minutes, *P* = .11) (Figure 3).

**TABLE 2:** Procedural outcomes: ablation for cardiac arrhythmias using RMN

	Total	SVT	AT/AFL	AF	PVC/VT	*P*‐value
	n = 821	n = 46	n = 37	n = 502	n = 236	
Procedure time, min	125.9 ± 54.6	68.7 ± 44.9	101.5 ± 43.1	143.5 ± 41.5	103.5 ± 64.4	<.0001
Clinical time, min	88.9 ± 42.6	39.3 ± 34.4	70.5 ± 37.6	106.3 ± 31.6	64.3 ± 44.2	<.0001
Mapping time, min	17.7 ± 11.1	12.0 ± 9.6	17.8 ± 14.3	15.6 ± 5.3	23.3 ± 16.6	<.0001
Total X‐ray time, min	5.3 ± 3.9	3.5 ± 4.5	3.6 ± 2.8	6.4 ± 3.5	3.7 ± 4.0	<.0001
Doctor's X‐ray time, min	3.7 ± 3.1	2.5 ± 3.6	2.4 ± 2.7	4.6 ± 3.0	2.2 ± 2.6	<.0001
Control room's X‐ray time, min	1.6 ± 1.8	1.0 ± 1.6	1.2 ± 0.9	1.8 ± 1.3	1.5 ± 2.6	<.0001
RF applications, n	51 ± 37	11 ± 11	31 ± 27	74 ± 27	13 ± 11	<.0001
RF time, min	29.3 ± 19.2	5.8 ± 4.5	18.3 ± 11.8	41.6 ± 13.0	9.4 ± 7.7	<.0001

*Note:* Values are mean ± SD.

Abbreviations: AF, atrial fibrillation; AFL, atrial flutter; AT, atrial tachycardia; PVC, premature ventricular contraction; RMN, remote magnetic navigation; SVT, supraventricular tachycardia; VT, ventricular tachycardia.

All the SVT and focal AT cases were successfully ablated with RMN. Electrical PVI was achieved in 99.0% of the studied AF procedures. Acute success reached 90.3% in PVC/VT procedures.

### 3.4 Learning curve

**FIGURE 4:**
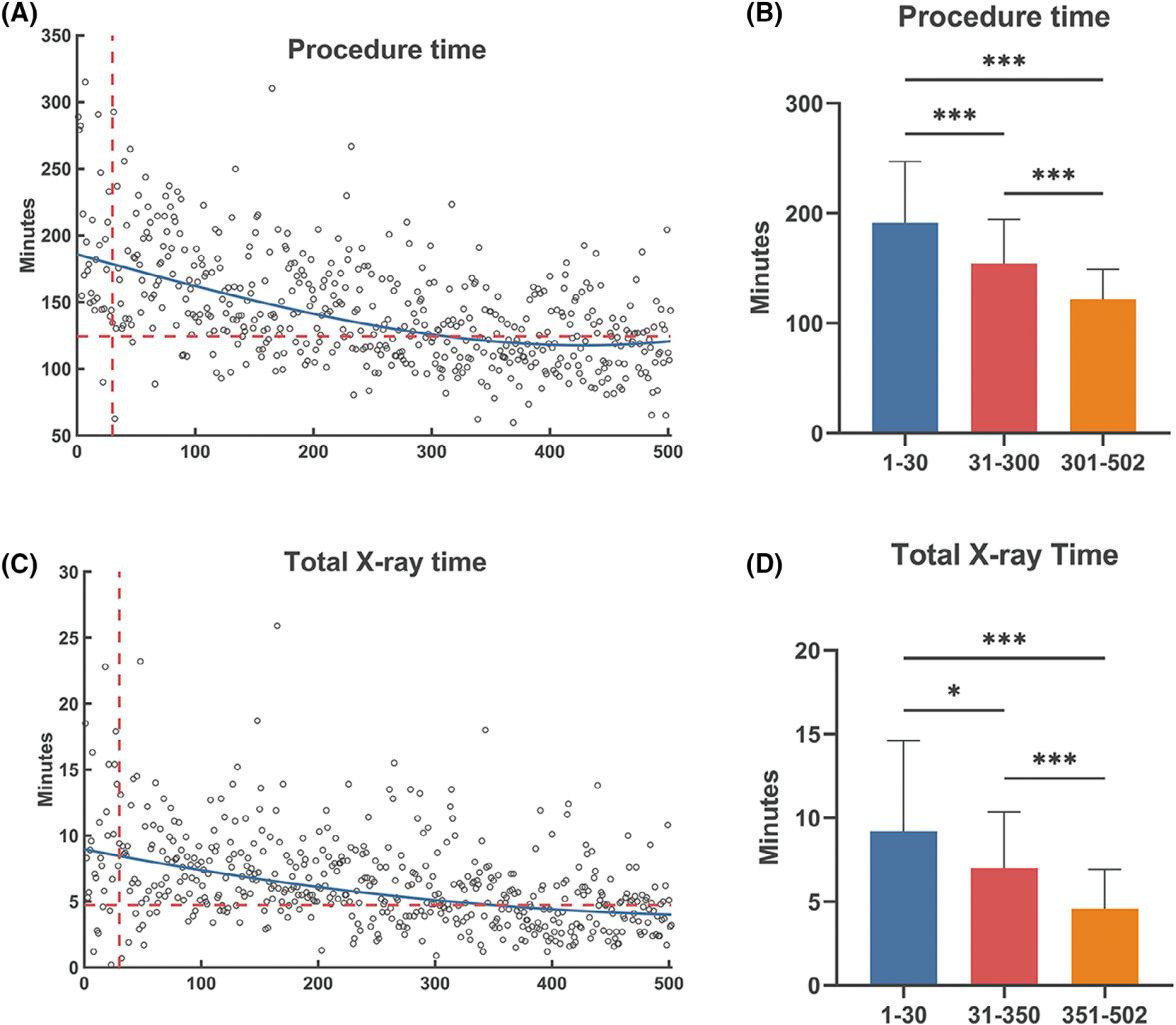
Procedure and total X‐ray time for all atrial fibrillation patient. Blue line: spline fit achieved by the least absolute residuals method. Transverse red dashed line: fitted time of the intercept point where the curve becomes flattened. Vertical red dashed line: the intercept point where the curve starts to drop significantly. A, Procedure time decreases along the learning curve and the curve becomes flat after 300 procedures. B, Procedure time decreased significantly from 191.4 ± 55.9 to 154.4 ± 39.8 and 121.7 ± 27.4 minutes, in P1‐3, respectively. C, Total X‐ray time reduces along the curve and the curve reach a plateau after 350 procedures. D, Total X‐ray time decreased from 9.2 ± 5.4 to 7.0 ± 3.4 and 4.6 ± 2.3 minutes, in P1‐3, respectively. ***P < .0001, *P < .05

An analysis was performed to determine the learning curve of the procedure and the total X‐ray time for consecutive AF ablation procedures by one operator and the results are shown in Figure 4A,C (*R* ^2^ = 0.97) and (*R* ^2^ = 0.98), respectively. The curves of procedure time and total X‐ray time started to decrease from the initial 30 procedures, and reached a plateau after 300 and 350 procedures, respectively, where both regression coefficients became indistinguishable from zero (*P* = .46and *P* = .87). Comparisons of the procedure and total X‐ray time among three phases are given in Figure 4B,D. Procedure time decreased significantly from 191.4 ± 55.9 to 154.4 ± 39.8 and 121.7 ± 27.4 minutes, in P1‐3, respectively. Similarly, total X‐ray time decreased from 9.2 ± 5.4 to 7.0 ± 3.4 and 4.6 ± 2.3 minutes, in P1‐3, respectively.

## 4 DISCUSSION

### 4.1 Major findings

To our knowledge, this is the first large‐scale study reporting the safety, the learning curve, and the procedural outcomes among different types of arrhythmias for RMN‐guided ablation in a single center. The main findings of this study are as follows. RMN‐guided ablation is safe, as verified by the low overall complication rate (0.5%) and reduced X‐ray time. The learning curve of RMN is relatively short. Even at the very beginning of the study, low procedure time and total X‐ray time could be achieved and procedure efficiency could be improved along the learning curve.

### 4.2 Learning curve of RMN‐guided ablation

In this study, AF cases, with the largest sample size, were used to demonstrate the effect of the learning curve on RMN‐guided ablation procedure outcomes. To our knowledge, this might be the first large‐scale study regarding RMN learning curve. We have established that as the experience grows, both procedure time and total X‐ray time decreases significantly. Previously, Pappone et al^12^ assessed the changes in procedure outcomes of RMN‐guided ablation in 40 patients with AF. They found that compared with the first 12 patients, the ablation time and fluoroscopy time of the last 28 patients shortened considerably. It suggested RMN ablation might have a steeper learning curve, but limited by sample size. Our large‐scale single‐center data provide evidence that the curve of clinical time and total X‐ray time dropped quickly, and flattened after about 300 cases. The effect of the learning curve on procedure efficiency has been demonstrated. The complication rate in our series is remarkably low; therefore, in general, RMN can be quickly adopted by electrophysiologists without the cost of compromised safety.

### 4.3 Safety of RMN‐guided ablation

Earlier studies have demonstrated that compared with manual procedures, RMN‐guided ablation has a lower complication rate.^13^, ^14^ However, previous reports mostly focused on a single type of arrhythmia. In this retrospective study, 1003 cases were analyzed and arrhythmia types included SVT, AT, AFL, AF, PVC, and VT. The overall complication rate was 0.5% and the major complication rate was even lower (0.1%). Only one case underwent cardiac tamponade, and no procedure‐related death occurred. Our data demonstrated the general safety of RMN ablation for all above‐mentioned arrhythmias. A recent epidemiological study that calculated the incidence of complications in patients who underwent catheter ablation from 2000 to 2013 in the United States reported that the total incidence of in‐hospital peri‐procedural complications was 5.46%, and VT ablation was associated with the highest rate of complication (9.90%).^15^ Of note, the safety endpoint in our study is independent of the type of arrhythmia ablated. The superior safety profile of RMN can be attributed to the following reasons. First, the floppy and steerable ablation catheter can achieve a relatively low tissue contact force, reducing the possibility of cardiac perforation. Second, the RMN system can maintain catheter stability despite changes in cardiac rhythm or cardiorespiratory movement, which remarkably reduces the incidence of pericardial tamponade.^16^, ^17^, ^18^ Hematoma or hemorrhage at access site is the most common complication of ablation.^15^ Compared with the manual procedure, the sheath of RMN system is fixed to the QuikCAS Cardiodrive system and remains relatively still with the femoral vein. This allows for fewer sheath maneuvers and can reduce endothelium injury and thrombosis at the tip of the sheath. Finally, the relatively large number of procedures in our center may also contribute to the low complication rate.

### 4.4 Fluoroscopy and procedure time of RMN‐guided ablation

Multiple studies have shown that exposure to radiation is associated with tumorigenesis among interventional cardiologists.^19^, ^20^ Meanwhile, studies comparing RMN and manual ablation have consistently reported that application of RMN can reduce radiation exposure.^21^, ^22^ A recent meta‐analysis also reported a significant reduction in fluoroscopy time.^23^ The mean total X‐ray time (5.3 ± 3.9 minutes) was remarkably low and X‐ray time continued to decrease as the learning curve traversed. Moreover, we noted that differences in X‐ray time among ablations for differing types of arrhythmias was more related to doctor's X‐ray time than to control room's X‐ray time. This finding further supports that when the operator is in the control room, utilizing the RMN system, radiation exposure can be kept low regardless of the type of arrhythmia ablated.

There are conflicting data regarding procedure time of RMN‐guided ablation. Some^24^ have reported prolonged procedure time while others^25^ have disagreed, reporting lower and desirable procedure time. Even published meta‐analyses have reached differing conclusions, possibly due to dissimilar inclusion criteria.^26^, ^27^ The heterogeneity between studies regarding procedure time may be influenced by studies completed by different generations of RMN, during an extended time period in which globally, there was neither understanding nor consensus regarding optimal application of the technology. Additionally, when compared with conventional ablation and other RMN studies, procedure time is relatively low in this study. This may be related to the effect of the learning curve, increased use of RMN best practices, and large sample size. Although our data are promising, larger randomized controlled trials are needed to demonstrate the superiority of RMN over traditional manual pull‐wire catheter navigation, for the ablation treatment of cardiac arrhythmias.

## 5 LIMITATIONS

This is a single‐center retrospective observational study. Second, in AF procedures, additional ablations such as linear ablation and CAFÉ ablation, which could potentially influence the procedural outcomes, were not analyzed. Third, this was not a controlled study to compare the procedural outcomes with conventional manual procedures.

## 6 CONCLUSIONS

RMN‐guided ablation is safe, which is confirmed by a very low overall complication rate and reduced X‐ray time. The procedure and X‐ray times decreased along the learning curve of RMN.

## CONFLICT OF INTEREST

The authors declare no potential conflict of interest.
